# THE EFFECT OF CHRONIC CONDITIONS ON DEPRESSIVE SYMPTOMS IS MODERATED BY SEXUAL MINORITY STATUS

**DOI:** 10.1093/geroni/igad104.2574

**Published:** 2023-12-21

**Authors:** Em Trubits, Jason Newsom, Jennifer Saucedo, Anda Botoseneanu, Heather Allore, Corey Nagel, David Dorr, Ana Quiñones

**Affiliations:** Portland State University, Portland, Oregon, United States; Portland State University, Portland, Oregon, United States; Portland State University, Portland, Oregon, United States; University of Michigan, Ann Arbor, Michigan, United States; Yale University, New Haven, Connecticut, United States; University of Arkansas for Medical Sciences, Little Rock, Arkansas, United States; Oregon Health & Science University, Portland, Oregon, United States; Oregon Health & Science University, Portland, Oregon, United States

## Abstract

It is important to examine disparities in the effects of physical health on depression among sexual minority (SM) older adults. We examined whether the effects of chronic conditions (CCs) and self-rated health (SRH) on depressive symptoms were moderated by sexual orientation among 3,741 adults aged 50+ (SM = 216) who participated in the 2016 wave of the Health and Retirement Study (HRS) and responded to the recently added sexual orientation item. Structural equation modeling was used to model the effects of SRH and CCs on depressive symptoms, while controlling for participant sex, age, years of education, net-worth, and race/ethnicity. Greater numbers of CCs were associated with greater depressive symptoms (b= .24, SE = .04, p <.001), whereas better SRH was associated with fewer depressive symptoms (b = -.60, SE = .05, p <.001). The direct effect of SM on depressive symptoms was significant, b = 1.14, SE = .48, p =.02, indicating greater depressive symptoms among SM adults. The interaction between SM and SRH on depressive symptoms was significant (b = -.39, SE = .17, p = .02). The same effect was seen for CCs (b = -.32, SE = .15, p = .04). Simple slopes revealed that for heterosexual older adults, greater CCs were associated with greater depressive symptoms (b = .27, SE = .04, p <.001), however this association was not significant for SM older adults (b = -.05, SE = .16, p = .75). Future research should examine health disparities among SMs in relation to mental health.

